# The Influence of UV Varnishes on the Content of Cysteine and Methionine in Women Nail Plates—Chromatographic Studies

**DOI:** 10.3390/ijms222212447

**Published:** 2021-11-18

**Authors:** Kamila Borowczyk, Rafał Głowacki

**Affiliations:** Department of Environmental Chemistry, Faculty of Chemistry, University of Łódź, 163 Pomorska Str., 90-236 Łódź, Poland; rafal.glowacki@chemia.uni.lodz.pl

**Keywords:** nail plate degradation, hybrid manicure, cysteine, methionine, high performance liquid chromatography

## Abstract

The main purpose of this work was to determine if the use of hybrid nail polishes causes changes in concentration of the most important sulfur amino acids that build nail plate structures, cysteine and methionine. We found that the average contents of cysteine and methionine in studied samples before the use of hybrid manicure were 1275.3 ± 145.9 nmol mg^−1^ and 111.7 ± 23.8 nmol mg^−1^, respectively. After six months of hybrid manicure use, the average amount of these sulfur amino acids in studied samples were 22.1% and 36.5% lower in the case of cysteine and methionine, respectively. The average amounts of cysteine and methionine in nail plate samples after the use of hybrid manicures were 992.4 ± 96.2 nmol mg^−1^ and 70.9 ± 14.8 nmol mg^−1^, respectively. We also confirmed that in studied women the application of UV light varnishes reduced the thickness of the nail plate, from 0.50 ± 0.12 mm before to 0.46 ± 0.12 mm after the use of the hybrid manicure.

## 1. Introduction

Nails are products of the epidermis, built mainly with keratins, and which are composed of up to 22% cysteine (Cys) residues [1]. The nail plate is formed as a result of the division of the cells forming the matrix of the nail. Its spatial orientation causes the nails to grow parallel to the surface of the finger. The nail plate is located directly on the bearing, which is tasked with the nourishing of the nail [2]. Its shape is an individual feature that depends mainly on the shape of the distal phalanges of a given individual [1]. It is built mainly from hard keratin and lipids. Histologically, it consists of three layers: dorsal, intermediate and ventral [3]. The upper surface of the plate in healthy individuals has a bright pink shape and a slight gloss, and the density and thickness increase towards the free edge of the nail even by 8.8% [4]. The thickness of nails increases with age and ranges from 0.5 to 0.7 mm. It is higher in people who do physical work and in men [5]. The average growth rate of the plate is from 1.9 to 4.4 mm monthly and is determined by many factors, including gender, age and season. Full regrowth of the hand nail plate takes from 90 to 150 days [4].

The primary function of nails in mammals is to protect the fingertips from mechanical damage. In addition, the nails facilitate precise finger movements, increase tactile sensitivity and the ability to grasp [6]. Nails develop around the 10th week of fetal life and continue to grow throughout life. The permanent growth of nail plates results from the constant keratosis of cells which do not become exfoliated [4].

An important factor affecting the physical properties of nails is the degree of hydration. Despite the high resistance to external factors, frequent hand washing and disinfection, which is especially important in current times, can increase the flexibility and fragility of nails. In contrast, the dehydration of tiles caused by excessive use of cosmetic products, especially acetone and detergents, can lead to splitting, dryness and brittleness in the plate [7].

Methionine (Met) and Cys are sulfuric amino acids present in nail plate keratins. These compounds also form structures of other proteins. Sulfur amino acids play many important roles in the human body; they can protect cells from free radicals and oxidative stress, and are responsible for stiffening the protein helix [8]. Met is involved in methylation reactions. It is necessary for normal growth and repair of damaged tissues. In addition, it affects the skin’s elasticity, as well as the strength of hair and nails [9]. Cys is an endogenous thiol amino acid formed as a product of catabolism of proteins in the transsulfuration reaction by converting Met to homocysteine and further to Cys [10]. The presence of Cys has been confirmed in hair, nails and skin. It plays an important role in the production of collagen and keratins and is responsible for their durability and flexibility [9].

Keratin is a heterogeneous protein which has a three-dimensional structure (α-helix, β-harmonica or random). Its structure is about 60% hydrophobic and 40% hydrophilic chemical groups [11]. It has very high mechanical and chemical resistance. It is not soluble in water and is characterized by high stability. The specific properties of this protein are related to the large number of Cys and disulfide bonds present in its structure [12]. Keratin contains a large amount of glycine, valine, and serine; however, tryptophan, lysine and Met can be also found in higher levels. Cys, in the form of sulfide or disulfide, is present in keratins in the largest content, usually from 7 to 12% of total amino acids. This compound is responsible for the formation of covalent bonds in the protein structure [11]. The higher number of -S-S- bonds makes keratins harder, stiffer and less stretchy [12].

The resistance of keratin to chemical and physical factors makes its decomposition difficult. The degradation of keratin can be caused by acids, alkalis, elevated temperature, and UV radiation, because in its structure there are fragments which absorb UV radiation [12].

A healthy nail plate is characterized by a bright pink color, a smooth, shiny, slightly convex surface and an oblong shape. Any visible changes in the nail structure should be shared with a doctor, because they may indicate a number of serious systemic diseases including psoriasis, renal failure, hyperthyroidism, iron deficiency, liver cirrhosis, etc. [13]. The damage to nails does not always result from disease; they are often the result of mechanical damage, improper care, the use of toxic substances, allergies and fungal diseases. Another frequent cause of nail issues is a popular method of nail coloring based on UV varnishes.

In recent years, the method of decorating nails, using a hybrid manicure, has become very popular. It is a cosmetic procedure in which UV hybrid varnish is applied to a previously fumed and degreased nail plate. Lacquers require additional curing with a UV light lamp. An acetone or mechanical treatment are required to remove old layers of the polish. Application of the hybrid nail polish ensures the aesthetic appearance of the hands, allows women to hide distorting changes in the nails, increases their psychological comfort and saves them time. On the other hand, the use of UV radiation to cure the varnish, as well as the drastic way of polishing, can cause significant damage to the nail plates.

The main goal of this work was to study the impact of the use of UV hybrid manicures on the content of sulfur amino acids, such as Cys and Met in human nail plates, and their health and aesthetic appearance. For the determination of these compounds in nail plate samples, methods based on an high performance liquid chromatography (HPLC) with spectrophotometric and spectrofluorimetric detection were applied. These previously published analytical protocols were developed and validated in our laboratory.

## 2. Results and Discussion

### 2.1. Acidic Hydrolysis of Nail Plate Samples

#### 2.1.1. The Influence of Time on the Hydrolysis Yield of Nail Plate Samples

**Figure 1:**
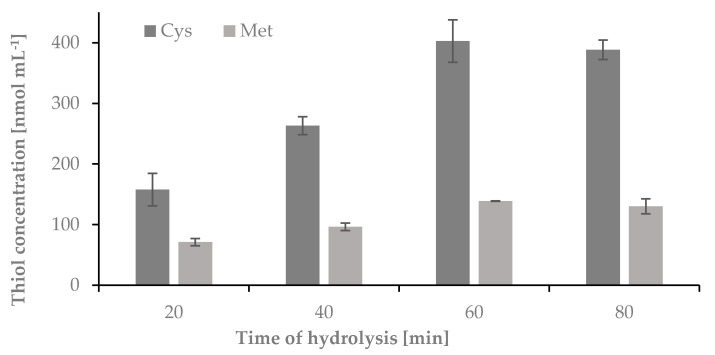
The influence of time on the hydrolysis yield of nail plate samples, measured as signals of cysteine-2-chloro-1-methylquinolinium tetrafluoroborate and methionine-*o*-phthaldialdehyde-N-acetylcysteine derivatives; *n* = 3.

Under acidic or alkaline conditions, peptide and disulfide bonds are broken. Acid hydrolysis is carried out using mainly mineral acids, often at elevated temperatures. The solution after hydrolysis contains free amino acids. The first signs of keratin degradation are noticeable after 30 s of heating at 185 °C. The process is carried out in an aqueous solution. As a result, oligopolymers, peptides and amino acids are formed [11]. To release Cys and Met from nail plate keratins, acidic hydrolysis of the sample was required. For this purpose HCl was used [14,15]. In order to establish optimum conditions for the sample, hydrolysis time and temperature as well as concentration and volume of HCl were tested. In the first step of this research, the optimal time of hydrolysis was studied. The hydrolysis was carried out in 1-mL Wheaton Gold Band ampoules using 5 mg of nail plate sample and 100 µL of 6 mol L^-1^ HCl. The ampoules were sealed under flame, and the samples were hydrolyzed at 120 °C for 20, 40, 60 and 80 min. After each time point the ampoules were centrifuged for 1 min at 10,000 rpm and opened. 50 μL of the hydrolyzed mixture was transferred from the glass ampoules to 0.5 mL Eppendorf tubes and evaporated over 20 min to dryness in a thermostat at 100 °C, dissolved in 50 μL of water, and treated to obtain cysteine-2-chloro-1-methylquinolinium tetrafluoroborate (Cys-CMQT) and methionine-*o*-phthaldialdehyde-N-acetylcysteine (Met-OPA-NAC) derivatives. The obtained results have been presented in Figure 1.

The highest concentration of Cys and Met were observed in samples hydrolyzed over 60 and 80 min. This study indicated that complete hydrolysis of the sample occurred after 60 min.

#### 2.1.2. The Influence of Temperature on the Hydrolysis Yield of Nail Plate Samples

**Figure 2:**
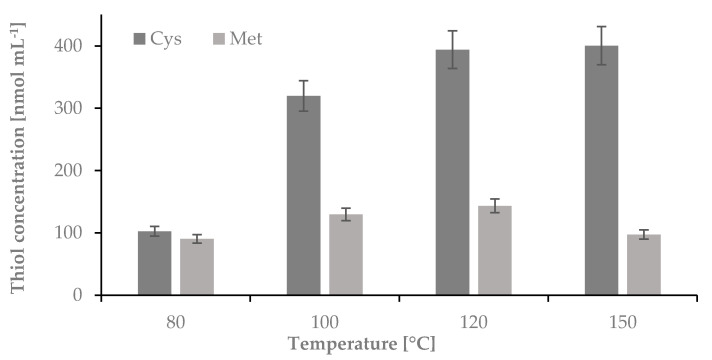
The influence of temperature on the hydrolysis yield of nail plate samples, measured as signals of cysteine-2-chloro-1-methylquinolinium tetrafluoroborate and methionine-*o*-phthaldialdehyde-N-acetylcysteine derivatives; *n* = 3.

As the next step of the study, the temperature of hydrolysis was optimized. In this case, the hydrolysis was carried out in 1-mL Wheaton Gold Band ampoules using 5 mg of nail plate sample and 100 µL of 6 mol L^−1^ HCl. The samples were incubated at 80, 100, 120 and 150 °C for 60 min. After the time the ampoules were centrifuged for 1 min at 10,000 rpm and opened. 50 μL of the hydrolyzed mixture was transferred from the glass ampoules to 0.5 mL Eppendorf tubes and evaporated over 20 min to dryness in a thermostat at 100 °C, dissolved in 50 μL of water, and treated to obtain Cys-CMQT and Met-OPA-NAC derivatives (see Figure 2).

All amino acids are crystalline substances with high melting points, which in most cases correspond to decomposition temperatures of these compounds. Most often, these temperatures are over 200 °C [9]. Our temperature test ranged from 80 to 150 °C. The experiments have shown that for Cys temperatures higher than 150 °C can be used without changing the total concentration of the compound in the hydrolyzed mixture. However, in the case of Met, we have observed more than a 30% reduction in the amount of this compound after incubation at 150 °C in the hydrolyzed mixture. As it was proven in previously published studies, under acidic conditions in temperatures above 120 °C, Met can be converted to homocysteine thiolactone. However, in our studies we did not detect homocysteine thiolactone, we confirmed that the hydrolysis of nail plate samples dedicated for determination of Met must be performed at temperatures lower than 150 °C. To carry out the next step of our studies we decided to perform the hydrolysis at 120 °C [16].

#### 2.1.3. The Influence of Concentration of HCl on the Hydrolysis Yield of Nail Plate Samples

**Figure 3:**
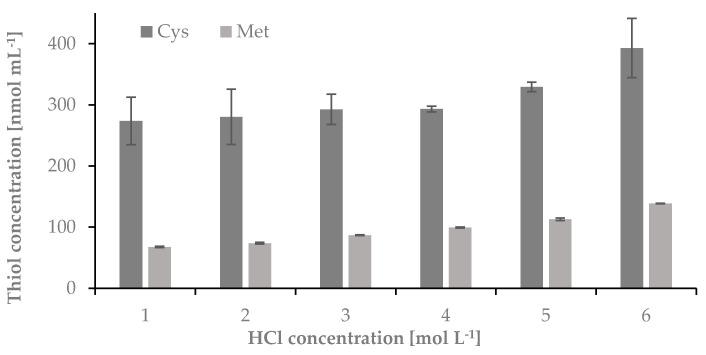
The influence of HCl concentration on the hydrolysis yield of nail plate samples, measured as signals of cysteine-2-chloro-1-methylquinolinium tetrafluoroborate and methionine-*o*-phthaldialdehyde-N-acetylcysteine derivatives; *n* = 3.

Next, a concentration of HCl used for hydrolysis was optimized. In this case, the hydrolysis was carried out in 1-mL Wheaton Gold Band ampoules using 5 mg of nail plate sample and 100 µL of 1, 2, 3, 4, 5, and 6 mol L^−1^ HCl. The samples were incubated at 120 °C for 60 min. Next, the ampoules were centrifuged for 1 min at 10,000 rpm and opened. 50 μL of the hydrolyzed mixture was transferred from the glass ampoules to 0.5 mL Eppendorf tubes and evaporated over 20 min to dryness in a thermostat at 120 °C, dissolved in 50 μL of water, and treated to obtain Cys-CMQT and Met-OPA-NAC derivatives. The results have been presented in Figure 3.

#### 2.1.4. The Influence of a Volume of HCl on the Hydrolysis Yield of Nail Plate Samples

**Figure 4:**
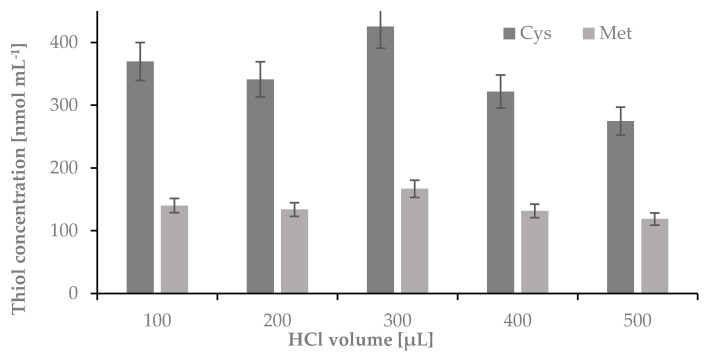
The influence of volume of 6 mol L^−1^ HCl on the hydrolysis yield of nail plate samples, measured as signals of cysteine-2-chloro-1-methylquinolinium tetrafluoroborate and methionine-*o*-phthaldialdehyde-N-acetylcysteine derivatives; *n* = 3.

Finally, a volume of HCl used for hydrolysis was optimized. As previously, the hydrolysis was carried out in 1-mL Wheaton Gold Band ampoules using 5 mg of nail plate sample and 100, 200, 300, 400 or 500 µL o f 6 mol L^−1^ HCl. Hydrolysis was performed using parameters established in previous experiments. The samples were incubated at 120 °C for 60 min. After this time the ampoules were centrifuged for 1 min at 10,000 rpm and opened. Half of the volume of the acid used for hydrolysis was transferred from the glass ampoules to 0.5 mL Eppendorf tubes and evaporated to dryness in a thermostat at 120 °C, dissolved in 50 μL of water, and treated to obtain Cys-CMQT and Met-OPA-NAC derivatives (see Figure 4).

The optimization of the HCl volume applied for hydrolysis indicated that to obtain the highest yield of the process 300 μL of the acid must be used.

### 2.2. Reduction and Derivatization

#### 2.2.1. Reduction of the Disulfide Bonds

The Cys and N-acetylcysteine (NAC) needed for Met derivatization are mainly present in oxidative form. For this reason a reduction reaction is required to release the thiol groups and make them available for the derivatizing reagent. *Tris-*(2-carboxyethyl) phosphine (TCEP) is known to be powerful, also under mild in terms of of pH and temperature [17,18,19,20], and for these reasons was applied in our studies.

#### 2.2.2. Derivatization of a Thiol Group

**Figure 5:**
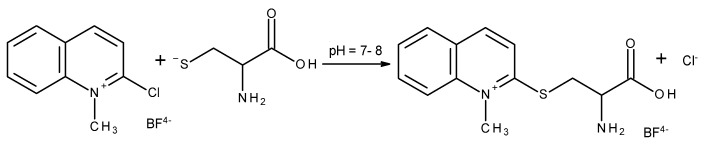
The scheme of the derivatization reaction of cysteine with the use of 2-chloro-1-methylquinolinium tetrafluoroborate.

2-Chloro-1-methylquinolinium tetrafluoroborate (CMQT) is a commonly used derivatization reagent dedicated to the determination of endogenous and exogenous thiols in biological samples by using the HPLC-UV technique [21,22,23]. For this reason, to continue studies on the influence of UV varnishes on the content of Cys and Met in female nail plates the derivatization reaction between Cys and CMQT was chosen. The scheme of the reaction is presented in Figure 5.

**Figure 6:**
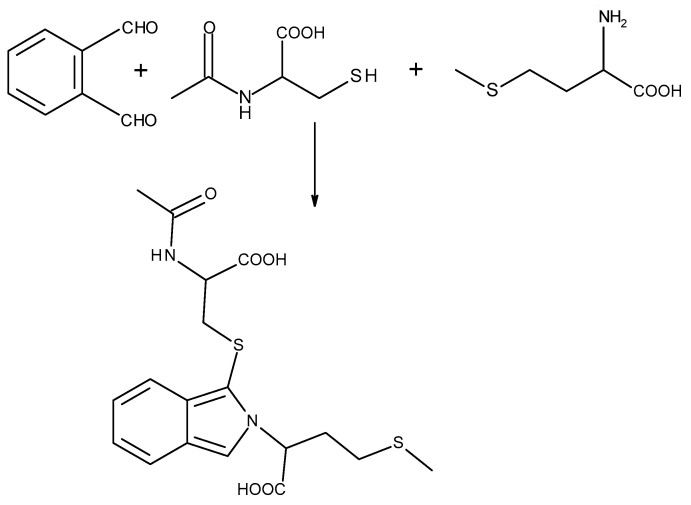
The scheme of chemical derivatization reaction of methionine with *o*-phthaldialdehyde and N-acetylcysteine.

To control the concentration of Met in nail plate samples, a method based on simultaneous derivatization of Met with NAC and o-phthaldialdehyde OPA and separation of the derivative was applied. The scheme of the derivatization reaction of Met with NAC and OPA has been presented in Figure 6. This method was previously developed in our laboratory and successfully applied for biological sample analysis [24].

**Figure 7:**
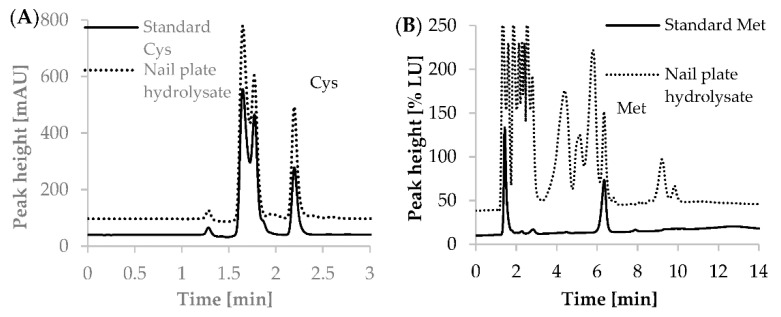
Representative chromatograms for Cys in human nail plate hydrolysates after reduction with TCEP and derivatization with CMQT (**A**) and for Met in human nail plate hydrolysates after on-column derivatization with OPA and NAC (**B**). Chromatographic conditions as described in Section 3.2.4 and Section 3.2.5.

The representative chromatograms of nail plate hydrolysates for Cys and Met have been presented in Figure 7.

#### 2.2.3. Selectivity and Specificity

CMQT is a highly selective and specific derivatizing reagent dedicated for the derivatization of thiol groups. The thiol—CMQT reaction gives us better selectivity, since only peaks of thiols are seen on a chromatogram [21]. Similar properties can be observed in case of sulfur amino acids, in which the derivatization reaction with OPA can be performed in the presence of both thiol and amino groups. For Met and OPA derivatives detected with the use of a spectrofluorimetric detector, the selectivity and specificity are also determined by the detection parameters [24]. In the case of human nail plate samples, thiols such as glutathione, NAC, α-lipoic acid or cysteine-glycine do not exist in keratins, and for this reason an investigation of these compounds was not required. However, to confirm selectivity of the applied methods, we tested six individual hydrolysates of human nail plate samples collected from six women. In the case of Cys, this was done by considering the potential interferences at the region assigned to chromatographic peaks of homocysteine. This compound possesses a thiol group which reacts with CMQT and might be present in keratin structures [14,15]. In the case of Met the study of selectivity was obtained by investigating the potential interference at the region assigned to chromatographic peaks of the homocysteine and OPA derivatives. Cys was not observed under the studied conditions. The selectivity and specificity studies were performed using the assays procedure and HPLC conditions described in Section 3.2.3, Section 3.2.4, Section 3.2.5 and Section 3.2.6.

#### 2.2.4. Matrix Effects

In chromatographic analyses, the researchers can be faced with the matrix effect in which the analytical method might have been effected by matrix components. In the presented study the matrix effects were investigated during the revalidation and implementation of the previously developed methods [21,24]. The evaluation of the matrix effect concerned the comparing of calibration curves in multiple sources of the hydrolysates of human nail plate samples against a calibration curve in the pooled matrix. The performed study indicated that calibration curves created from a pooled matrix did not differ significantly from the ones prepared in samples from six individual sources. In particular, the slope of the regression lines did not differ more than 3.7% for Cys and 2.8% for Met. The obtained results indicated the absence of any matrix effect and confirmed that most of the interfering matrix components were eliminated during optimization of sample preparation protocols. In case of applied methods the matrix effect might have been eliminated by the dilution of acidic hydrolysates.

#### 2.2.5. Method Calibrations

To establish concentrations of Cys and Met in the hydrolyzed mixture, calibrations of both methods were required. Samples of the hydrolyzed mixture, spiked with standard solutions and dedicated for calibration analyses were prepared using the same protocols of derivatization and HPLC analysis as samples of hydrolysates of nail plates before and after applying a hybrid manicure. For Cys and Met, six-point calibration plots were constructed. For preparation of calibration curves in human nail plates, hydrolysates in 10 μL-aliquots of samples were spiked with the increasing amounts of standard solutions of Met and cystine (Cys)_2_ to provide final concentrations of exogenous Cys as follows: 50; 100; 200; 300; 400; 600 nmol mL^−1^ hydrolysate and final concentrations of exogenous Met as follows: 60; 80; 120; 160; 200; 250 nmol mL^−1^ hydrolysate. The calibration standard solutions were prepared in triplicates. The calibration curves have been presented in Supplementation data (Figures S1 and S2). As shown in Figure 7, the Cys and Met derivatives were eluted after 2.26 (±0.01; *n* = 5) min and 6.32 (±0.02; *n* = 5) min, respectively. The coefficients of variation of retention time of the Cys and Met derivatives were 0.24% and 0.26%, respectively. The coefficients of variation of peak area for analyzed substances in studied ranges of concentrations were from 0.36 to 2.59% for Cys and from 0.1 to 11.6% for Met.

The calibration curves were obtained by plotting the peak areas against the analyte concentrations. Regression equations, correlation coefficients as well as methods imprecision and recovery have been presented in Table 1.

During recalibration of the previously published methods applied for the determination of Cys and Met we prepared new calibration curves and calculated intra-and inter-day precision and accuracy of these assays. The procedures followed the guidelines for biological sample analysis [25,26]. The parameters of precision and accuracy were calculated using the results of the analysis of human nail plate hydrolysate samples spiked with known amounts of Cys and Met and were analyzed in triplicates for both intra-day and inter-day measurements. Precision was expressed in terms of the coefficient of variation, whereas accuracy was considered as the percentage of analyte recovery calculated by expressing the mean measured amount as a percentage of the added amount. To calculate these parameters, the additional calibration curves obtained on that occasion were used. The detailed data have been presented in Table 2.

### 2.3. Cys and Met in Nail Plates before and after Applying a Hybrid Manicure

#### 2.3.1. The Content of Cys and Met in Nail Plates

The proper conditions of our nail plates are important to all of us. The appearance of hands, and above all nails, is not only our showcase, but also a reflection of the health of our body. Healthy nails should have a smooth, shiny surface and a light pink color. The most common causes of weakening the conditions of the nails are excessively aggressive cosmetic procedures, such as the excessive use of varnishes and solvents, and frequent micro-injuries caused during mechanical removing of UV light varnishes. The keratin found in nails is usually very hard, which is due to the presence of a significant amount of sulfur amino acids in the structure of the protein, including Cys and Met. Keratin is rich in Cys residues which have the ability to form strong covalent disulfide bridges. The presence of these bonds causes the structure of the entire protein to stiffen, making it hard and elastic. In addition, the stabilized structure makes the nails resistant to the action of proteolytic enzymes and insoluble in water. When the number of disulfide bridges in the structure of keratins decreases, the nails become more brittle and breakable, and thus more susceptible to damage. Impairing of the nail plates resulting from the use of gel and hybrid varnishes is a common phenomenon. Damage to nail plates occurs particularly during improper performance of the procedure and mechanical removal of the varnish, which leads to traumatic onycholysis. This is due to the fact that the adhesion of hybrid varnish is stronger than that of the plate to the nail bed [27].

The main goal of our work was to confirm whether the use of a UV hybrid manicure affects the content of sulfur amino acids, such as Cys and Met in nail plates, and their health and aesthetic appearance. To study the hypothesis we measured the concentration of Cys and Met in nail plates collected from 10 women without earlier using a hybrid manicure and after six months of application of these type of nail cosmetics.

**Figure 8:**
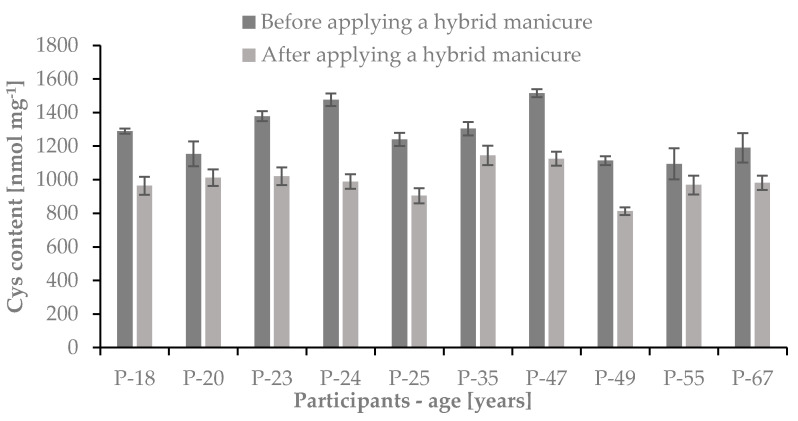
The Cys content in the nail plates of studied women before and after the use of hybrid manicure; *n* = 3.

**Figure 9:**
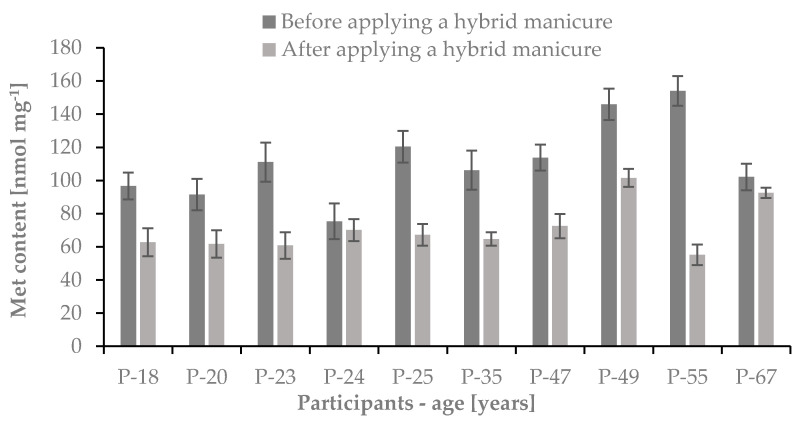
The Met content in the nail plates of studied women before and after the use of hybrid manicure; *n* = 3.

We have found that the average contents of Cys and Met in studied samples before the use of a hybrid manicure were 1275.3 ± 145.9 nmol mg^−1^ and 111.7 ± 23.8 nmol mg^−1^, respectively. After six months of the use of a hybrid manicure the average contents of these sulfur amino acids in studied samples were 22.1% lower in the case of Cys and 36.5% lower in the case of Met. The average contents of Cys and Met in nail plate samples after the use of hybrid manicure were 992.4 ± 96.2 nmol mg^−1^ and 70.9 ± 14.8 nmol mg^−1^, respectively. The obtained results have been presented in Figure 8 and Figure 9.

**Figure 10:**
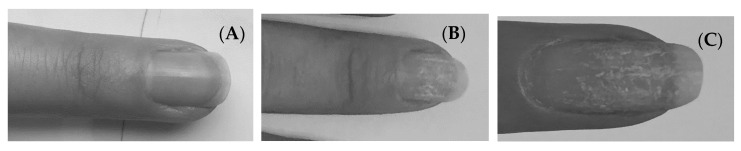
A woman’s nail plate before (**A**) and after (**B**,**C**) the use of hybrid manicure.

Our findings indicate that gel and hybrid manicures cause damage to the nail plate base, which becomes rough and shows destruction of disulfide bridges. It was also observed during application of new layers of the UV light varnish that white streaks appeared on the plate (see Figure 10). This type of discoloration, called medicinia or nail vitiligo, is caused by keratin degranulation. It usually disappears within a few weeks, but in severe cases it may persist until the nail is completely fused. There are also reports of psoriatic lesions manifested by excessive peeling of the skin under the nail during long-term use of gel and hybrid manicures [27]. Patch tests revealed a contact allergy caused by acrylates. The effects of matting the plate and removing the varnish by soaking in acetone are also visible.

#### 2.3.2. Measurement of Nail Plate Thickness

One of the stages of hybrid manicure is matting the nail plate, which consists in grinding the top layer of the nail with fine-grained files and polishes. In order to investigate the changes in the thickness of the nail plate, this parameter was measured before and after applying the hybrid manicure to all participants of the study. For this purpose, a digital caliper by DIGI-MET with a range of 150 mm and measurement accuracy of 0.01 mm was used.

**Figure 11:**
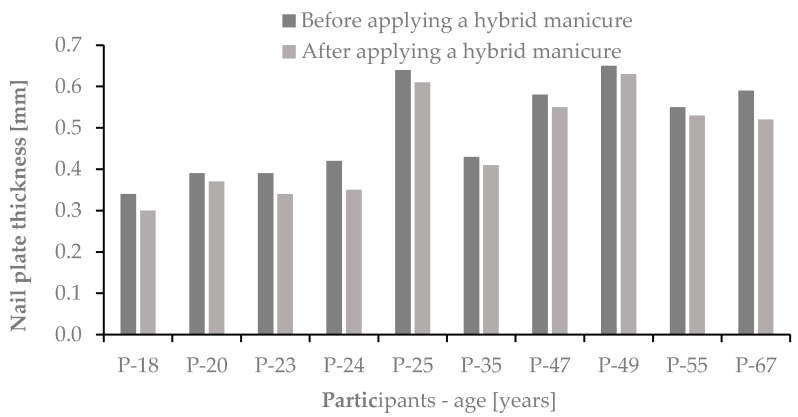
Changes in the thickness of the nail plate before and after applying a hybrid manicure.

We confirmed that in studied women the application of UV light varnishes reduced the thickness of the nail plate from 0.50 ± 0.12 mm before to 0.46 ± 0.12 mm after the use of the hybrid manicure. The results are shown in Figure 11.

People who use this type of manicure struggle with the fragility and sagging of the plate for a long time. The nails show an area where the plate becomes thinner, and redness can often be noticed due to the greater visibility of blood vessels; this defect is called a sagging nail plate [27]. Our findings have proven the previously published observations.

## 3. Materials and Methods

### 3.1. Materials

#### 3.1.1. Chemicals

Cystine (Cys)_2_, methionine (Met), N-acetylcysteine (NAC), *o*-phthaldialdehyde (OPA) and *tris-*(2-carboxyethyl)phosphine (TCEP) were received from Sigma Aldrich Company (St. Louis, MO, USA). HPLC gradient grade acetonitrile (MeCN), hydrochloric acid (HCl), sodium hydrogen phosphate heptahydrate (Na_2_HPO_4_·7H_2_O), sodium dihydrogen phosphate dihydrate (NaH_2_PO_4_·2H_2_O) and sodium hydroxide (NaOH) were from J.T. Baker (Deventer, The Netherlands). Perchloric acid (PCA) and trichloroacetic acid (TCA) were from Merck (Darmstadt, Germany). The derivatization reagent, 2-chloro-1-methylquinolinium tetrafluoroborate (CMQT) was synthesized in our laboratory [28]. Deionized water was produced in our laboratory.

#### 3.1.2. Instrumentation

For the determination of Met and Cys previously described methods were used [21,24]. All analyses were performed on an HPLC systems (Agilent Technologies, Waldbronn, Germany) equipped with a quaternary pump, vacuum degasser, autosampler, module of on-column derivatization temperature control, spectrophotometric detector Series 1100 and spectrofluorometric detector 1200 Series. All chromatographic analyses were controlled by HP ChemStation software. Cys and Met were separated using reversed-phase, chromatographic columns Zorbax SB-C18 (155 × 4.6 mm, 5 Å) and Hamilton PRP-1 (150 × 4.6 mm, 5 μm) (Energy Way, Reno, NV, USA), respectively. Water was purified using Milli-QRG system (Millipore, Vienna, Austria). For pH measurement an HI 221 (Hanna Instruments, Woonsocket, RI, USA) pH meter was used. Hydrolysis of the samples were performed in Grant thermostat.

#### 3.1.3. Stock Solutions

Stock solution of (Cys)_2_ 0.05 mol L^−1^ was prepared in 0.1 mol L^−1^ hydrochloric acid. Stock solution of Met 0.1 mol L^−1^ was prepared in 0.1 mol L^−1^ hydrochloric acid. CMQT 0.1 mol L^−1^ was prepared in water. All solutions were kept at 4°C for several days. The working solutions were prepared by dilution with water as needed, and processed immediately. Fresh stock solutions of TCEP (0.25 mol L^−1^) were prepared in 0.2 mol L^−1^ pH 7.4 phosphate buffer before the disulfide bonds reduction. NAC (0.5 mol L^−1^) and OPA (0.01 mol L^−1^ in NaOH 0.1 mol L^−1^) were prepared daily.

### 3.2. Sample Protocols

#### 3.2.1. Human Nail Plate Samples

Nail samples were collected from a group of 10 women aged from 18 to 67 years. The samples were collected before starting the use of a hybrid manicure and after six months from the initiation of this procedure. None of those women had received any type of manicure before the start of the study. The biological material was stored in closed 1.5 mL Eppendorf tubes at room temperature in the darkness.

Informed consent was obtained from all subjects involved in the study. Written informed consent has been obtained from the patients to publish this paper. The study was conducted according to the guidelines of the Declaration of Helsinki, and approved by the Ethics Committee of University of Lodz (protocol code 18/KBBN-UŁ/I/2016 approved on 14 April 2016).

#### 3.2.2. Hydrolysis of Nail Plate Samples

To release the amino acids from nail keratins an acid hydrolysis was carried out [14,15]. To perform the experiments, 5 mg of nails were washed with 200 μL of mixture methanol and deionized water (1:1; *v*:*v*) and dried with a paper towel. This process was repeated twice. The samples were placed in 1 mL glass ampoules and 300 μL of 6 mol L^−1^ HCl solution was added. The glass ampoules were closed in the flame of the gas burner and placed in a thermostat. The acidic hydrolysis was carried out at 120 °C for 60 min. After this time, the closed ampoules were centrifuged for 1 min, at 10,000 rpm and opened. 150 μL of the hydrolyzed mixture was transferred from the glass ampoules to 0.5 mL Eppendorf tubes and evaporated over 20 min to dryness in a thermostat at 100 °C. The obtained precipitate was stored at −20 °C to further analytical procedures.

#### 3.2.3. Sample Preparation for Determination of Cys

The obtained precipitate of nail samples hydrolysate was dissolved in 150 μL of deionized water and used for HPLC-UV and HPLC-FLD analyses. Samples were prepared directly in HPLC vials. 10 µL of the hydrolysate was diluted with 180 µL of 0.2 mol L^−1^ (pH 7.8) phosphate buffer, treated with 5 µL of TCEP (0.25 mol L^−1^ in phosphate buffer) and kept for 10 min at room temperature. Next, the same volume of the derivatizing agent solution (5 µL 0.1 mol L^−1^ CMQT) was added. The derivatization reaction was carried out over 5 min at room temperature. To stop the derivatization reaction, 25 μL of 3 mol L^−1^ PCA was added. Next, an appropriate amount of deionized water was added to obtain the final volume of 500 µL. 10 μL of the final sample was injected into the chromatographic column.

#### 3.2.4. Sample Preparation for Determination of Met

The obtained precipitate of nail samples after the acidic hydrolysis was dissolved in 150 μL of deionized water. For the determination of Met, 10 μL of the hydrolysate was mixed with 100 μL of phosphate buffer (0.2 mol L^−1^; pH 7.4) and 50 μL of 0.5 mol L^−1^ NAC. In the next step the sample was treated with 5 μL of 0.25 mol L^−1^ TCEP in order to prevent oxidation of NAC involved in the derivatization reaction. The mixture was diluted with deionized water up to the final volume of 200 µL. 5 μL was injected into the chromatographic column. The derivatization reaction was carried out directly in the chromatographic column concurrent to the process of chromatographic separation.

#### 3.2.5. HPLC Conditions for Determination of Cys

The Cys-CMQT derivative was eluted by a mobile phase containing 65% of TCA (0.1 mol L^−1^; pH = 1.67) and 35% of MeCN with isocratic elution. Flow rate of the mobile phase was 1 mL min^−1^. For detection of Cys-CMQT the 355 nm wavelength was used. Separation was performer at room temperature over 4 min. Identification of the Cys-CMQT peak was based on the comparison of retention time and UV spectra of the signals with corresponding data obtained for authentic compound after derivatization with CMQT.

#### 3.2.6. HPLC Conditions for Determination of Met

For chromatographic separation of the Met-OPA-NAC derivative in acidic hydrolysate of nail plates, a reversed-phase PRP-1 column was used. The analyte was eluted by a mobile phase containing 0.01 mol L^−1^ OPA in 0.1 mol L^−1^ NaOH (A) and MeCN (B) in the gradient mode as follows: 0–8 min, 14–25% (B); 8–12 min, 25% (B), 12–14 min, 25–14% (B). The flow rate of the mobile phase was 1 mL min^−1^. For Met-OPA-NAC, a detection excitation at 348 nm and emission at 438 nm was used. Separation was performed at room temperature. Identification of Met-OPA-NAC peak was based on comparison of retention time and excitation and emission spectra with the corresponding set of data obtained for Met after derivatization with OPA and NAC [24].

## 4. Conclusions

Our research clearly indicates that the content of both Cys and Met in nail samples subjected to hydrolysis in each of the examined persons decreased significantly after six months of applying the “hybrid manicure”. The reduction in the amount of sulfur amino acids in the nail plates may be caused by deterioration of keratin formation. This is probably determined by the harmful influence of chemical, physical and mechanical agents. The nails have become more prone to dissolution, resulting in reduced resistance to chemical agents. The degradation of keratin can be caused by the use of harmful solvents and UV radiation. Additionally, a reduction in the thickness of nail plates after using a hybrid manicure was observed. This can result from the mechanical matting of the nail surface and the application of mechanical milling machines used to remove the layer of lacquer adhering to the nail plate.

## Figures and Tables

**Table 1:** Validation data.

	Linear Range [nmol mL^−1^]	Regression Equation	R^2^	Imprecision [%]		Recovery [%]	
				Min.	Max.	Min.	Max.
Cys	50.0–600.0	y = 0.131x + 60.39	0.999	0.4	2.6	100.9	122.2
Met	60.0–250.0	y = 1.008x + 74.4	0.998	0.1	11.7	95.0	109.6

**Table 2:** Accuracy and precision.

	Concentrations	Precision [%]		Accuracy [%]	
		Intra-Day	Inter-Day	Intra-Day	Inter-Day
Cys [nmol mL^−1^]	50.0	4.1	5.6	100.3	112.2
	300.0	4.7	9.2	110.4	101.6
	600.0	2.3	8.1	93.2	98.1
Met [nmol mL^−1^]	60.0	3.7	3.6	99.3	115.6
	160.0	3.3	6.8	91.7	109.4
	250.0	5.3	5.6	99.8	104.4

## Data Availability

Not applicable.

## Supplementary Materials

The following are available online at https://www.mdpi.com/article/10.3390/ijms222212447/s1, Figure S1: Calibration curve of Cys in human nail plates hydrolysates, Figure S2: Calibration curve of Met in human nail plates hydrolysates.

## References

[1] Saleah S.A., Kim P., Seong D., Wijesinghe R.E., Jeon M., Kim J. (2021). A preliminary study of post-progressive nail-art effects on in vivo nail plate using optical coherence tomography-based intensity profiling assessment. Sci. Rep..

[2] Chen A.F., Chimento S.M., Hu S., Sanchez M., Zaiac M., Tosti A. (2012). Nail damage from gel polish manicure. J. Cosmet. Dermatol..

[3] Wang B., Yang W., McKittrick J., Meyers M.A. (2016). Keratin: Structure, mechanical properties, occurrence in biological organisms, and efforts at bioinspiration. Prog. Mater. Sci..

[4] de Berker D.A.R., Andre J., Baran R. (2007). Nail biology and nail science. Int. J. Cosmet. Sci..

[5] Barba C., Méndez S., Martí M., Parra L., Coderch L. (2009). Water content of hair and nails. Thermochim. Acta.

[6] Shipp L.R., Warner C.A., Rueggeberg F.A. (2014). Further investigation into the risk of skin cancer associated with the use of UV nail lamps. JAMA Dermatol..

[7] Batory M., Wołowiec-Korecka E., Rotsztejn H. (2019). The effect of various primers improving adhesiveness of gel polish hybrids on pH, TOWL and overall nail plates condition. J. Cosmet. Dermatol..

[8] Brosnan J.T., Brosnan M.E. (2006). The sulfur-containing amino acids: An overview. J. Nutr..

[9] Baran R., Schoon D. (2004). Nail beauty. J. Cosmet. Dermatol..

[10] Iorizzo M., Piraccini B.M., Tosti A. (2007). Nail cosmetics in nail disorders. J. Cosmet. Dermatol..

[11] Shah K.N., Rubin A.I. (2012). Nail disorders as signs of pediatric systemic disease. Curr. Probl. Pediatr. Adolesc. Health Care.

[12] Sun C., Koppel K., Adhikari K. (2015). Sensory factors affecting female consumers acceptability of nail polish. Int. J. Cosmet. Sci..

[13] Singal A., Arora R. (2015). Nail as a window of systemic diseases. Indian Dermatol. Online J..

[14] Borowczyk K., Wróblewski J., Suliburska J., Akahoshi N., Ishii I., Jakubowski H. (2018). Mutations in homocysteine metabolism genes increase keratin, N-homocysteinylation and damage in mice. Int. J. Genom..

[15] Borowczyk K., Suliburska J., Jakubowski H. (2018). Demethylation of methionine and keratin damage in human hair. Amino Acids.

[16] Butz L.W., Du Vigneaud V. (1932). The formation of a homologue of cystine by the decomposition of methionine with sulfuric acid. J. Biol. Chem..

[17] Kuśmierek K., Chwatko G., Głowacki R., Kubalczyk P., Bald E. (2011). Ultraviolet derivatization of low-molecular-mass thiols for high performance liquid chromatography and capillary electrophoresis analysis. J. Chromatogr. B.

[18] Borowczyk K., Krawczyk M., Kubalczyk P., Chwatko G. (2015). Determination of lipoic acid in biological samples. Bioanalysis.

[19] Kuśmierek K., Chwatko G., Głowacki R., Bald E. (2009). Determination of endogenous thiols and thiol drugs in urine by HPLC with ultraviolet detection. J. Chromatogr. B Analyt. Technol. Biomed. Life Sci..

[20] Borowczyk K., Olejarz P., Chwatko G., Szylberg M., Głowacki R. (2020). A simplified method for simultaneous determination of α-lipoic acid and low-molecular-mass thiols in human plasma. Int. J. Mol. Sci..

[21] Bald E., Głowacki R., Drzewoski J. (2001). Determination by liquid chromatography of free and total cysteine in human urine in the form of its S-quinolinium derivative. J. Chromatogr. A.

[22] Borowczyk K., Wyszczelska-Rokiel M., Kubalczyk P., Głowacki R. (2015). Simultaneous determination of albumin and low-molecular-mass thiols in plasma by HPLC with UV detection. J. Chromatogr. B.

[23] Kamińska A., Głowacka I.E., Pasternak B., Głowacki R., Chwatko G. (2019). The first method for determination of lipoyllysine in human urine after oral lipoic acid supplementation. Bioanalysis.

[24] Borowczyk K., Chwatko G., Kubalczyk P., Jakubowski H., Kubalska J., Głowacki R. (2016). Simultaneous determination of methionine and homocysteine by on column derivatization with o-phtaldialdehyde. Talanta.

[25] European Medicines Agency Committee for Medicinal Products for Human Use, Guideline on Bioanalytical Method Validation. https://www.ema.europa.eu.

[26] FDA Guidance for Industry Bioanalytical Method Validation. https://www.fda.gov.

[27] Motswaledi M.H., Mayayise M.C. (2010). Nail changes in systemic diseases. SA Fam. Pract..

[28] Bald E., Głowacki R. (2001). 2-Chloro-1-methylquinolinium tetrafluoroborate as an effective and thiol specific UV-tagging reagent for liquid chromatography. J. Liq. Chrom. Relat. Technol..

