# Supplementary material for: The Influence of UV Varnishes on the Content of Cysteine and Methionine in Women Nail Plates—Chromatographic Studies
Source: Int J Mol Sci. 2021 Nov 18;22(22):12447. doi: 10.3390/ijms222212447 (PMC8625047; doi:10.3390/ijms222212447)
Supplement: Supplementary file 1 [file ijms-22-12447-s001.zip › ijms-1450027-supplementary.pdf]

International Journal of
Molecular Sciences

MDPI

Article

The influence of UV varnishes on the content of cysteine and methionine in women nail plates - chromatographic studies

Kamila Borowczyk*, **Rafał Głowacki**

Department of Environmental Chemistry, Faculty of Chemistry, University of Łódź, 163 Pomorska Str., 90-236 Łódź, Poland; kamila.borowczyk@chemia.uni.lodz.pl

* Correspondence: kamila.borowczyk@chemia.uni.lodz.pl; Tel.: +48-42-635-58-44

Citation: Borowczyk, K.; Głowacki, R. The Influence of UV Varnishes on the Content of Cysteine and Methionine inWomen Nail Plates—Chromatographic Studies. *Int. J. Mol. Sci.* **2021**, *22*, 12447. https://doi.org/10.3390/ijms222212447

Academic Editor: María A. Pajares

Received: 20 October 2021
Accepted: 15 November 2021
Published: 18 November 2021

Publisher's Note: MDPI stays neutral with regard to jurisdictional claims in published maps and institutional affiliations.

Copyright: © 2021 by the authors. Licensee MDPI, Basel, Switzerland. This article is an open access article distributed under the terms and conditions of the Creative Commons Attribution (CC BY) license (http://creativecommons.org/licenses/by/4.0/).

Abstract: The main purpose of this work was to indicate if the use of hybrid nail polishes causes changes of concentration of the most important sulfur amino acids such as cysteine and methionine, that build nail plates structures. The second aim was to estimate a correlation between concentration of sulfur amino acids in nails and their weakening, fragility and thinness. We found, that the average contents of cysteine and methionine in studied samples before the use of hybrid manicure were 1275.3 ± 145.9 nmol mg^{-1} and 111.7 ± 23.8 nmol mg^{-1}, respectively. After 6 months the use of hybrid manicure the average contents of these sulfur amino acids were in studied samples were 22.1% lower in case of cysteine and 36.5% lower in case of methionine. The average contents of cysteine and methionine in nail plate samples after the use of hybrid manicure were 992.4 ± 96.2 nmol mg^{-1} and 70.9 ± 14.8 nmol mg^{-1}, respectively. We also confirmed that in studied women the application of UV light varnishes reduced the thickness of the nail plate, from 0.50 ± 0.12 mm before to 0.46 ± 0.12 mm after the use of the hybrid manicure.

Keywords: nail plate degradation; hybrid manicure; cysteine, methionine, high performance liquid chromatography,

1. Supplementary data

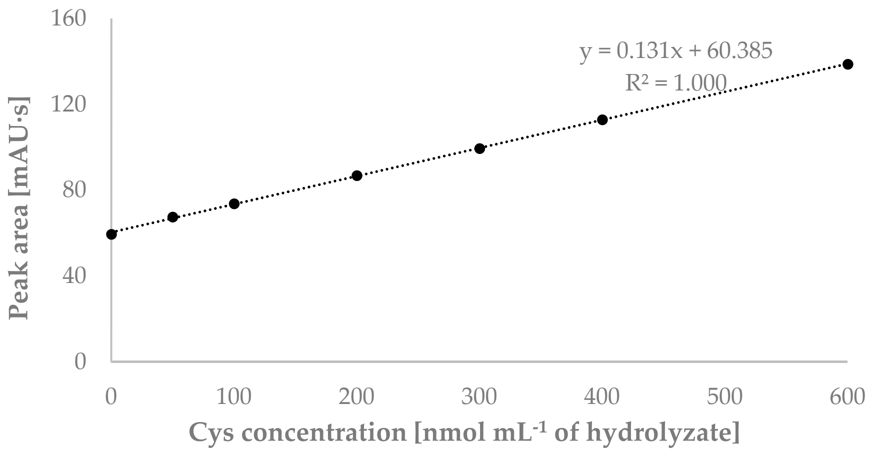

Fig. S1. Calibration curve of Cys in human nail plates hydrolysates.

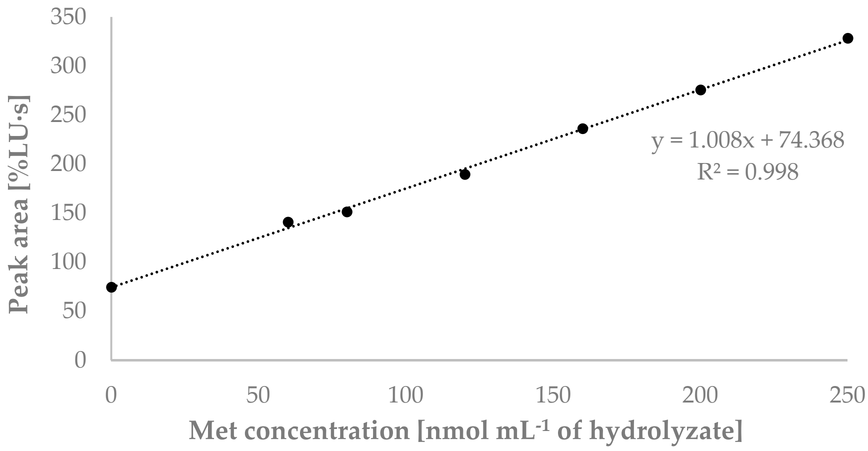

Fig. S2. Calibration curve of Met in human nail plates hydrolysates.
